# Colour vision in stomatopod crustaceans: more questions than answers

**DOI:** 10.1242/jeb.243699

**Published:** 2022-03-28

**Authors:** Amy Streets, Hayley England, Justin Marshall

**Affiliations:** Queensland Brain Institute, University of Queensland, St Lucia, QLD 4072, Australia

**Keywords:** Spectral sensitivity, Spectral discrimination, Photoreceptor, Plasticity, Invertebrate vision, Behaviour

## Abstract

Stomatopod crustaceans, or mantis shrimps, are known for their extensive range of spectral sensitivity but relatively poor spectral discrimination. Instead of the colour-opponent mechanism of other colour vision systems, the 12 narrow-band colour channels they possess may underlie a different method of colour processing. We investigated one hypothesis in which the photoreceptors are proposed to act as individual wave-band detectors, interpreting colour as a parallel pattern of photoreceptor activation, rather than a ratiometric comparison of individual signals. This different form of colour detection has been used to explain previous behavioural tests in which low-saturation blue was not discriminated from grey, potentially because of similar activation patterns. Results here, however, indicate that the stomatopod *Haptosquilla trispinosa* was able to easily distinguish several colours, including blue of both high and low saturation, from greys. The animals did show a decrease in performance over time in an artificially lit environment, indicating plasticity in colour discrimination ability. This rapid plasticity, most likely the result of a change in opsin (visual pigment) expression, has now been noted in several animal lineages (both invertebrate and vertebrate) and is a factor we suggest needs attention and potential re-examination in any colour-based behavioural tests. As for stomatopods, it remains unclear why they achieve poor colour discrimination using the most comprehensive set of spectral sensitivities in the animal kingdom and also what form of colour processing they may utilise.

## Abstract (precis)

**Summary:** Stomatopods are able to distinguish both high and low saturation colours from greys, but this ability decreases over time under artificial lighting, suggesting plasticity in their colour vision.

## INTRODUCTION

**Fig. 1.**
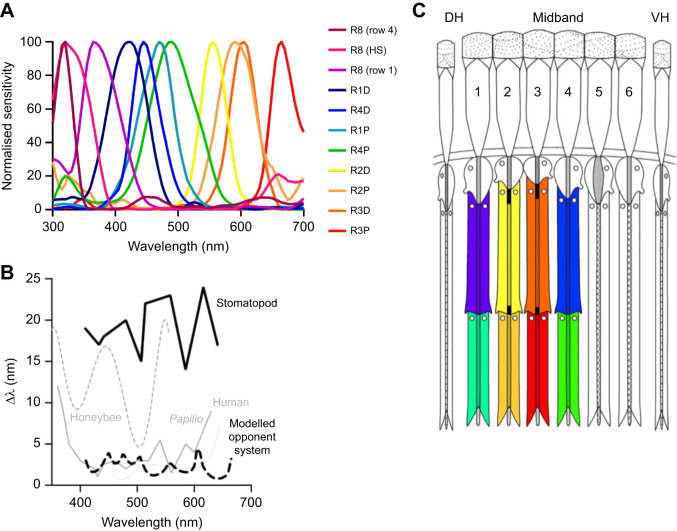
**Model of stomatopod (*Haptosquilla trispinosa*) visual system.** (A) Normalised electrophysiological response of each type of photoreceptor in the first four midband rows. (B) Spectral discrimination ability at different wavelengths (λ) for stomatopods and example animals. See Materials and Methods for discussion and references. (C) Stylized midband showing approximate photoreceptor-type sensitivity as colours. The distally placed UV-sensitive R8 cells are not coloured. Numbers refer to the respective midband rows and the colours indicate the approximate spectral sensitivity of each tier. A and B were adapted from Thoen et al. (2014); C was adapted from Marshall et al. (2007).

Mantis shrimp, or stomatopods, possess perhaps the most complex retina of all visual systems known (Marshall, 1988; Cronin and Marshall, 1989; Marshall et al., 2007; Marshall and Arikawa, 2014). With 12 spectral photoreceptors (and others for polarisation and intensity detection bringing the total number of input channels to 20), they outnumber, with the possible exception of butterflies (Arikawa, 2003; Chen et al., 2016; Marshall and Arikawa, 2014), the receptor diversity of other animals, which commonly have between two and four spectral sensitivities (Fig. 1A; Barlow, 1982; Kelber and Osorio, 2010). The 12 colour receptors are spread evenly through the spectrum, sampling from just below 300 nm to above 700 nm, but most likely do not construct the dodecahedral colour space they are capable of, as there are no known colour tasks in nature requiring this degree of scrutiny (Barlow, 1982; Kelber and Osorio, 2010; Marshall and Arikawa, 2014). Their sharply tuned photoreceptor set has been proposed to be effective in achieving colour constancy in the spectrally challenging marine environment (Osorio et al., 1997), but this idea remains hypothetical.

While early behavioural tests demonstrated colour vision in stomatopods based on the von Frisch colour from greys paradigm (Von Frisch, 1974; Marshall et al., 1996), more recent and more detailed wavelength discrimination experiments suggest that stomatopods lack fine spectral discrimination (Fig. 1B; Thoen et al., 2014). Based largely on anatomical evidence (Marshall, 1988; Marshall et al., 1991a, 1991b), a four spectral window opponent comparison was originally proposed in which different eye regions (rows of ommatidia in the mid-band region of the eye) analysed discrete zones of the 400–700 nm spectrum (Fig. 1C). This would still enable very fine spectral analysis, in particular as a result of the sharp sensitivities the eye achieves with serial filtering mechanisms (Marshall, 1988; Cronin and Marshall, 1989).

**Fig. 2.**
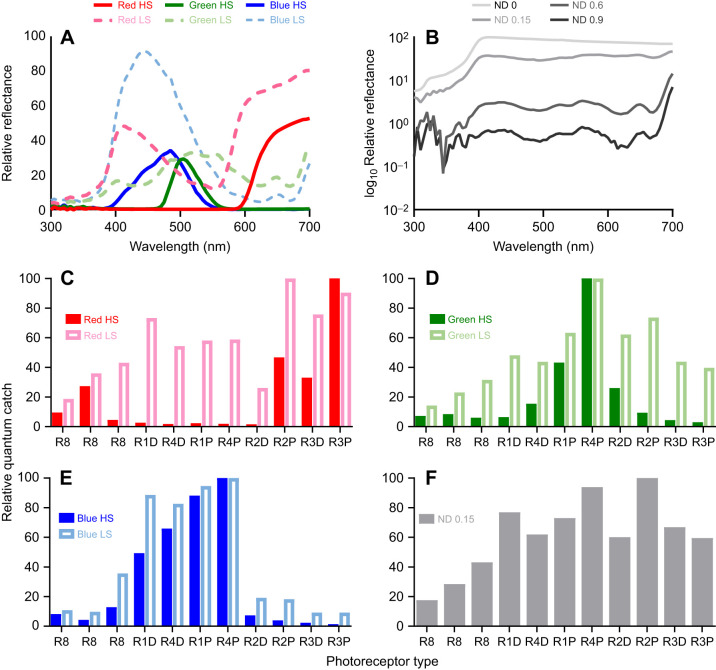
**Stimulus filters.** (A,B) Spectral reflectance measurements of (A) coloured filters and (B) neutral density (ND) filters. All curves have been normalized to ND 0 in order to demonstrate relative brightness. In A, highly saturated (HS) coloured filters are solid lines, and less saturated (LS) coloured filters are dashed. (C–F) Calculated relative quantum catch of each photoreceptor type to each coloured filter. Solid bars refer to highly saturated filters, and open bars to less saturated filters: (C) red, (D) green, (E) blue (less saturated blue is the same as that used in Marshall et al., 1996) and (F) ND 0.15. The horizontal axis gives photoreceptor types, including the R8 in row 4, the hemisphere and row 1, respectively (see Fig. 1).

Although the lack of fine spectral discrimination was surprising, these results did potentially provide an explanation for one of the observations made in the original ‘colours from grey’ behavioural assay. In this experiment, the peacock mantis shrimp, *Odontodactylus scyllarus*, could not discriminate a light blue feeding container from greys (Marshall et al., 1996). Upon analysing the response pattern of all colour photoreceptors, these authors found that the blue stimulus showed a similar photoreceptor activation pattern to the grey stimuli used as distractors in the behaviour paradigm (Fig. 2). In this case, the blue colour used was relatively under-saturated, or closer to grey, compared with the red, green and yellow. In an attempt to explain this failure in choice, it was suggested that mantis shrimp may analyse colour as a pattern of 12 excitations across the spectrum, rather than with any comparison of spectral sensitivity. This idea is congruent with their scanning eye movements as a way of examining coloured objects, it is expanded upon below (Marshall et al., 2014; Land et al., 1990; Land, 1999).

Stomatopod compound eyes are composed of two peripheral hemisphere regions on either side of a central midband. While the morphology of the ommatidia in the hemispheres is much like that in other crustaceans, the six rows of ommatidia in the midband are modified in a number of ways. It is in the top four midband rows that the specialisations for colour vision are found, each row being sensitive to three wavelength zones. The colour photoreceptors are sharpened and shifted in their spectral sensitivity by a number of filtering mechanisms including short wavelength filtering (in the UV) by the dioptric crystalline cone elements, photoreceptor tiering and, in rows 2 and 3, photostable colour filters (Bok et al., 2014; Cronin and Marshall, 1989; Marshall, 1988; Marshall et al., 2007) (Fig. 1A,C). Beneath the retina, information from the midband rows initially remains separated from the hemispheres, and through the eye-stalk neuropils, lamina, medulla and lobula, there are anatomically segregated zones that receive input from the midband and each of the hemispheres (Thoen et al., 2017). While the basic arrangement here appears similar to that in other arthropods, in fact centripetally there is increasing complexity and some cross-talk between retinal zones, including between the midband rows themselves (Thoen et al., 2018). Until effective electrophysiological recordings are made at these levels, further speculation is just that. Nonetheless, a brief review of past ideas, placed in the context of the knowledge level of their time, contributes to the background and motivation for the behavioural data presented here.

There are several hypotheses previously suggested to explain how mantis shrimps may process colour information. Based on initial anatomical evidence, it was originally proposed that photoreceptor signals outside the ultraviolet (UV) range, may be compared within each of the four colour-sensitive midband rows (1–4) (Fig. 1C). This intra-row comparison would deliver a possible ‘dichromatic’ opponent system, each row examining a limited spectral zone from 400 to 700 nm. Compelling evidence here is in the fact that the rhabdomeric cells in each tier of these rows are the same as those that, in other crustaceans and in the stomatopod hemispheres, are set up as polarisation opponent sub-populations, comparing, for example, horizontally polarised light with vertical (Glantz and Miller, 2002; Marshall et al., 1991a; Strausfeld and Nassel, 1981). This means that, without the need to reorganise existing wiring beneath the retina, a polarisation opponency becomes colour opponency.

An alternative idea is that stomatopods may analyse colour information in a manner similar to the processing of auditory information by the cochlea, examining the chromaticity of light within each spectral band as a continuum of frequency, rather than using opponent processing (Fig. 2C–F; Marshall et al., 2007). Instead of comparing spectral signals, downstream computation centres would determine colours by the pattern, or placement in the frequency continuum, of photoreceptor activation. This idea is sometimes called the ‘barcode’ hypothesis, likening the scanning over objects to other line-scan devices such as photocopiers, barcode readers or satellites (Wolpert, 2011). This idea has been used to explain the limited colour discrimination ability and argue for a system in some ways more like a colour categorising system based on which photoreceptors are activated (Marshall et al., 1996). Supporting evidence here comes in two forms. Firstly, there are striking similarities to the way colours are processed in the inferior temporal cortex of primates (Zaidi et al., 2014) and this may facilitate faster processing at the cost of poorer colour discrimination between similar wavelengths (Thoen et al., 2014). Secondly, the compressed optics of the stomatopod eye (Marshall and Land, 1993) drive the system to sample the world with slow scanning eye movements (Land et al., 1990). The barcode hypothesis also explains the apparent over-proliferation of spectral sensitivities and their narrow-band tuning, as this set of 12 is needed to cover the available spectrum from 300 to 700 nm (Marshall and Arikawa, 2014). As shown by Barlow (1982) and others, an opponent system only requires around four spectral sensitivities over this range to decode almost all colour information present on Earth.

There is in fact no reason why both opponency and some sort of pattern, barcode, categorical or frequency analysis system might not operate simultaneously. If stomatopods have divided up the spectrum into dichromatic bins, it may be that each of these is used to solve some sort of relevant task while an overall sense of colour is provided by barcode scanning. Having, in some ways, an over-precise opponent process has been suggested as a way to solve the problems of colour constancy underwater (Osorio et al., 1997). In order to further explore the various hypotheses, we conducted a series of experiments using a different species of stomatopod, *Haptosquilla trispinosa*, to determine whether all low-saturation colours, not just blue (Marshall et al., 1996), were more difficult to learn than high-saturation colours (experiment 1). If the barcode hypothesis were the only method of colour discrimination, we might expect the results of Marshall et al. (1996) to be repeated across the whole spectrum for the low-saturation colours and grey (Fig. 2C–F). If no comparisons, such as opponency, are made between photoreceptor types, then simply the activation of photoreceptors would cause low-saturation colours and greys to appear similar, and stomatopods would require high-saturation colours to respond to differences. Based on initial results, we also set out to examine any variability in performance, such as a change in visual performance, after being kept in captivity (experiment 2) and any innate preference for specific colours or colour types (experiment 3).

## MATERIALS AND METHODS

### Animal care

**Fig. 3.**
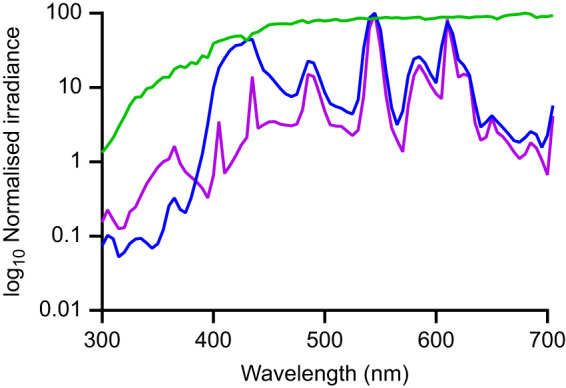
**Available light spectra.** Available light in the natural environment at Lizard Island Research Station (LIRS; green) and with overhead lights at the University of Queensland (UQ; experiment 1, blue; experiment 2, purple). Irradiance was normalized for each measurement to show relative spectral availability.

Stomatopods, *Haptosquilla trispinosa* (Dana 1852), were collected on the shallow reefs around Lizard Island Research Station (LIRS) (GBRMPA permit no. G17/38160.1) during August 2018, 2019 and 2020. They were individually housed in aquaria with small PVC tubes to use as burrows and fed small pieces of raw shrimp. Experiments requiring training were conducted either at the LIRS, under shaded but natural daylight, or at the University of Queensland (UQ), on a 12 h:12 h light:dark cycle with salinity between 32 and 35 ppm. The UQ aquarium lights consist of fluorescent tubes combined to provide illumination as close as possible to natural daylight (Fig. 3).

### Stimulus design

The stimuli were made of small white cable ties (2.5 mm width, 10 cm length), the ratchet-end of the cable tie making a convenient small feeding dish and flat end on which to attach various coloured or neutral density (ND) filters (Lee Filters, Andover, UK; see Templin, 2017, for further details). Filters were: high- and low-saturation red (Lee Filters 182/035, respectively), orange (287/162), green (124/725), blue (195/725) and ND filters (Lee Filters: ND 0, 0.15, 0.6, 0.9). The low-saturation blue filter (725) is the same as that used in Marshall et al. (1996). ND filters were also the same as those in Marshall et al. (1996) and other experiments (for review, see Kelber et al., 2003) to ensure the animals made choices based on colour, not brightness.

Fig. 2A,B shows spectral measurements of each stimulus filter attached to a cable tie. In order to make relevant comparisons, spectra were normalised to the maximum reflectance of the transparent filter ND 0. Photoreceptor responses were calculated using a modified quantum catch calculation (Kelber et al., 2003; Marshall et al., 1996). Individual spectral sensitivities for *H. trispinosa* were taken from Thoen et al., 2014 (Fig. 1A) and downwelling light was measured with an Ocean Optics USB2000 spectrophotometer (Fig. 3, experiment 1). These values were normalised by the individual spectra of the filters (Fig. 2A,B) to give the final photoreceptor response (Fig. 2C–F) as per Marshall and Vorobyev (2003).

### Experimental procedure

**Fig. 4.**
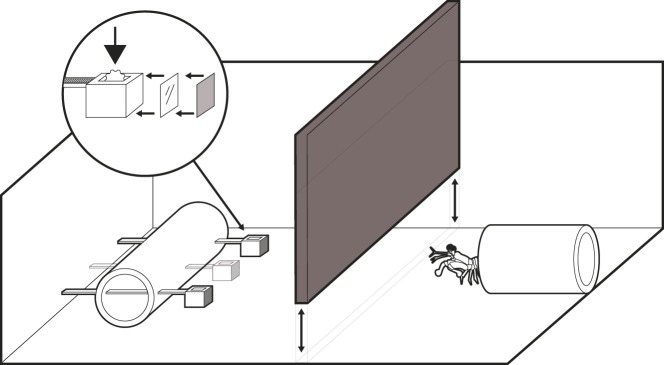
**Behavioural experiment.** The setup just before an experiment begins. The stomatopod is in its burrow when the barrier (dark grey) is inserted and the cable ties placed (either two or three, see Materials and Methods). The inset shows the cable tie setup: the filter (shaded) is stuck with double-sided tape (clear) onto front of the cable tie. Food is put into the cable tie during training trials (arrow). The removal of the barrier signals the start of a trial, and the stomatopod is then able to pull one cable tie into its burrow.

Feeding choice tests were used to compare the ability of *H. trispinosa* to discern either high- or low-saturation colours from neutral greys. Each stomatopod was assigned a single colour at high or low saturation: red, orange, green or blue. Animals readily emerged from their burrow to pick up the cable tie and then retreated back into their home to consume the food (Fig. 4). A successful choice was recorded if they took the correct cable tie first and pulled it toward their burrow.

Animals were introduced to their stimulus during a ‘priming’ week, where they were given a single cable tie, coloured according to their assignment, with food in the cavity created by the front of the cable tie. During the second week, they were ‘trained’ with a priming cable tie (containing food), plus the distractor(s) cable tie(s) (ND 0, 0.15, 0.6, 0.9) presented in a pseudo-random order. After the second week, the animals that reached approximately a 70% rate of cable tie feeding were used for testing. Animals that did not participate were considered untrainable and were removed. This method is further described in Templin (2017).

Fig. 4 shows the experimental setup. During a trial, each stomatopod was presented with their assigned colour and one (experiment 2) or two (experiment 1) ND distractors. Cable ties were held loosely in a holder so they could be easily removed. The cable ties were presented as a set, approximately 3 cm from the entrance to their burrow. In training trials, a small piece of food was placed inside the cavity of the target cable tie. During tests, the target cable tie and all ND distractors were newly fabricated and had never come into contact with food, in order to eliminate any residual olfactory cues.

Tests in which cable ties were not in contact with food were conducted once a day, 5 times a week. Additionally, mantis shrimp were trained once a week with food present to reinforce the behaviour. The stomatopods were given approximately 3 min to make a decision or were considered to not have participated. A trial began when the barrier inserted between the burrow and the stimulus (Fig. 4) was lifted. If the mantis shrimp chose correctly, it was rewarded with a small piece of food. If it chose the unrewarded colour, the cable ties were removed, and no food was awarded.

The combination of coloured cable ties and ND filters was made using a random number generator. Enough experiments were conducted so that each combination occurred at least once during all testing trials but not more than 3 times during both training and testing. The trials were ordered such that the target cable tie was not in the same location for two (for 3-choice trials, experiment 1) or three (for 2-choice trials, experiment 2) consecutive trials. If a mantis shrimp participated ≤2 times per week, for more than 1 week, it was considered untrainable, and therefore replaced.

### Individual experiments

#### Experiment 1: high/low saturation

In experiment 1, we investigated the original barcode hypothesis, the expectation being that the mantis shrimp might either find discrimination tests more difficult or fail them for all low-saturation colours. Animals were tested across eight colour types (four hues: red, orange, green and blue, each at two saturation levels: high and low). Stomatopods were given a 3-choice test, with two ND distractors. However, after a while it was noted that animals significantly preferred the middle position of the target cable tie (*P*<0.001), though no left/right bias was observed (*P*>0.5). Only one distractor was used in later experiments to prevent this bias, and in three-way choices, results were adjusted to account for this bias. These tests were exclusively conducted in captivity at UQ, beginning at least 3 months post-capture.

#### Experiment 2: high-saturation discrimination over time

In the second experiment, we tested whether the length of time in captivity affected the ability of the mantis shrimp to discriminate colour. Tests consisted only of highly saturated red, green and blue versus one ND distractor. Tests were divided into three time points to judge ability after different lengths of time spent in captivity: (1) 1 week at LIRS; (2) weeks 1–10 at UQ under artificial lighting; and (3) in the final weeks of testing (weeks 10–20) at UQ. Animals at LIRS were trained and tested for 1 week, 3 times a day.

#### Experiment 3: naive-choice tests

All naive-choice tests were conducted in the LIRS aquarium system under natural lighting in which freshly caught animals were given 24 h to acclimate. Two-choice tests were conducted across all colours (red, orange, green, blue): (A) high-saturation preference, (B) low-saturation preference, (C) preference for high or low saturation, and (D) preference for colour versus ND grey. In high- and low-saturation tests (A and B), individuals were given a random pair of red, orange, green or blue stimuli of the same saturation type. In the saturation preference test (C), individuals were given a high- and low-saturation stimulus of a single colour. Finally, for colour versus grey preference (D), animals were given a randomised colour (red, orange, green or blue) and neutral grey (ND 0, 0.15, 0.6 and 0.9) pair. Each individual was only used once in each experiment type; some were used in two different experiments because of collection restrictions. Individuals were given 30 min to make a choice. If an individual did not participate, it was tested again with the same choice for up to two additional sessions, and then replaced.

### Data analysis

Ability to learn the task was evaluated with a simple binomial test (BINOM.DIST, Microsoft Excel). This approach compares the number of times the mantis shrimp chose a particular stimulus with the number of times they would be expected to choose it by chance. Individuals were included in the analysis only if they were successful more often than expected by chance during training trials, as well as those that participated in at least 10 trials in experiment 1, and at least 3 trials at LIRS or 5 trials at UQ in experiment 2. Animals were assumed to have learned the task if they selected the target stimulus more often than chance. Experiment 3 analysis was also performed with a binomial test to determine significance between the two choices.

Data from experiments 1 and 2 were analysed using the general linear mixed effects model package in R (lme4 package, R version 3.5.3; Bates et al., 2011). Success for each experiment was used for the response variable in all analyses. Depending on the experiment, the colour (red, orange, green, blue), colour type (high or low saturation), time period, and ND distractors were treated as fixed factors. The individual ID of each animal was treated as a random factor in all analyses.

To understand interacting effects, including the effect of the ND filter types and cable tie position, a *post hoc* test was performed for individual colours and colour types using Tukey's multiple comparisons test (Hothorn et al., 2008). Only data from ‘test’ trials were used, and only when the stomatopod performed the task, in order to account for participation interruption due to moulting.

## RESULTS

### Experiment 1: high/low saturation

**Fig. 5.**
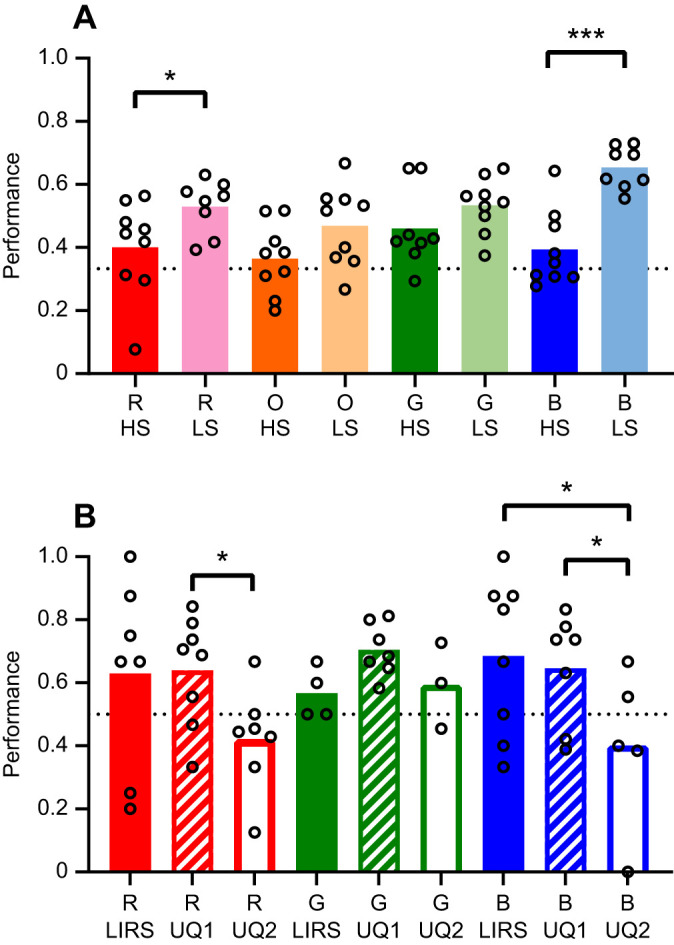
***Haptosquilla trispinosa* performance in captivity.** Average performance of individuals in each category. (A) Experiment 1 results: comparison of high-saturation (HS) and low-saturation (LS) stimuli. (B) Experiment 2 results: performance over time in captivity, at LIRS, and in the first 10 weeks (UQ1) and in the second 10 weeks (UQ2) at UQ. The dotted black line indicates the chance level. Circles indicate each individual's average performance. R, red; O, orange; G, green; B, blue. Significance: *P<0.05, **P<0.01, ***P<0.001.

*Haptosquilla trispinosa* were able to learn to distinguish all colours from grey (binomial test: highly saturated orange *P*<0.05 and highly saturated blue *P*<0.01; *P*<0.0001 for all others). There was an overall trend towards better performance with less saturated colours (Fig. 5A). This was significant for red (GLMM, *P*<0.05) and blue (*P*<0.0001), but not orange (*P*>0.05) or green (*P*>0.1) (Table 1).

**Table 1.**
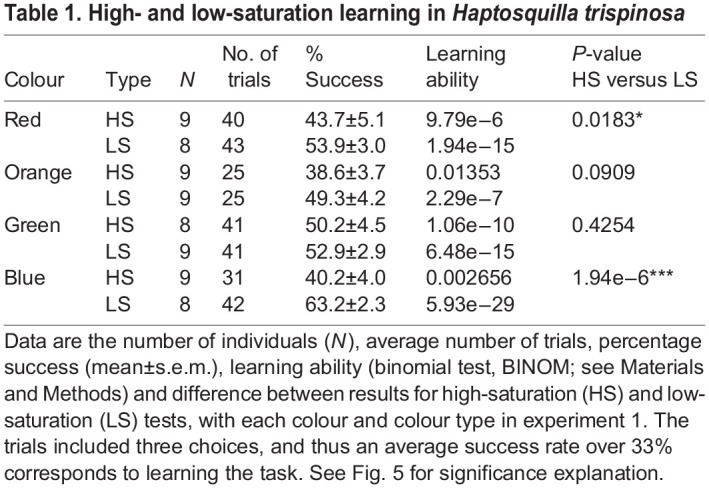
High- and low-saturation learning in *Haptosquilla trispinosa*

An average of 9 individuals per colour type participated in an average of 36 trials (range 10–71, depending on trainability and mortality). There was no significant variation between individuals (model variance <0.1, Fig. 5A). Overall, many animals were less successful in trials when at least one of the distractors was ND 0.15 and/or 0.6, especially in less saturated red and highly saturated green (Table S1A). These ND distractors were similar in brightness to the target stimuli (Fig. 2A,B). There was no significant effect of sex or size on the overall success; therefore, these factors were not included in further analysis (*P*>0.5).

### Experiment 2: discrimination over time

*Haptosquilla trispinosa* were trained to discern red, green and blue high-saturation stimuli from a neutral grey distractor, at LIRS and UQ. Over the first 10 week period in captivity (weeks 1–10), all animals were able to learn the task successfully (*P*<0.001). In the second 10 week period in captivity (weeks 11–20), accuracy was severely diminished. During this time, animals were not able to choose the coloured stimulus significantly more than chance for all colours (*P*>0.05) (Table 2).

**Table 2.**
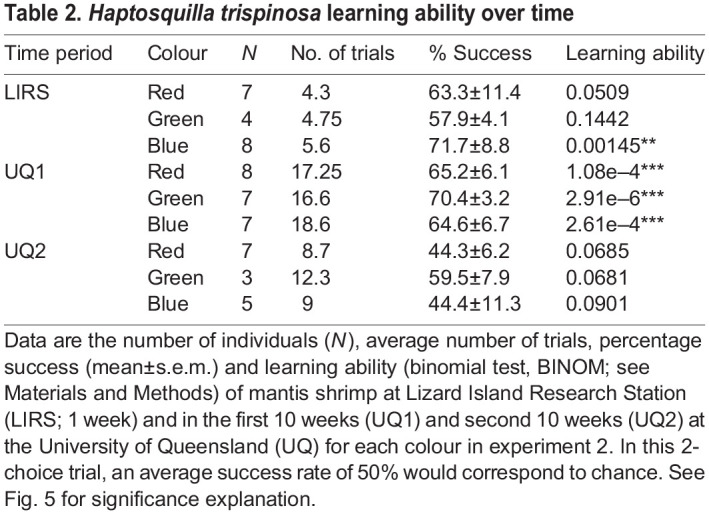
*Haptosquilla trispinosa* learning ability over time

For all colours, *H. trispinosa* became significantly less successful at selecting the correct stimuli after spending time in artificial light (Fig. 5B; GLMM *P*<0.001). After the first 10 weeks in captivity, animals became less effective at selecting red and blue high-saturation stimuli (*P*<0.05). In addition, animals learning highly saturated blue were more successful at LIRS in natural light than in the artificial lab light (*P*<0.05) (Fig. 5B, Table 3).

**Table 3.**
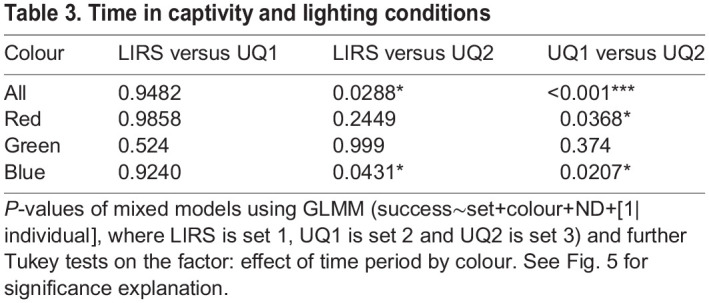
Time in captivity and lighting conditions

Individual variance in the linear mixed model was less than 0.2, and lower for most colours and time periods, except for highly saturated blue and at LIRS (approximately 0.5). On average, 6 individuals were used per colour with an average of 5 trials each at LIRS; 7 individuals per colour with 17 trials each during the first 10 weeks at UQ; and 5 individuals with 10 trials each during the second 10 weeks at UQ (Table 2). Animals participated significantly less in test trials during the second 10 weeks than in the first 10 weeks in the lab environment (*P*<0.0001). Animals did significantly better in trials with ND 0 (GLMM *P*<0.0001), when trained to green and blue (*P*<0.01 and *P*<0.05, respectively), and at LIRS and in the first 10 weeks at UQ (*P*<0.05 and *P*<0.0001, respectively) (Table 4; Table S1B).

**Table 4.**
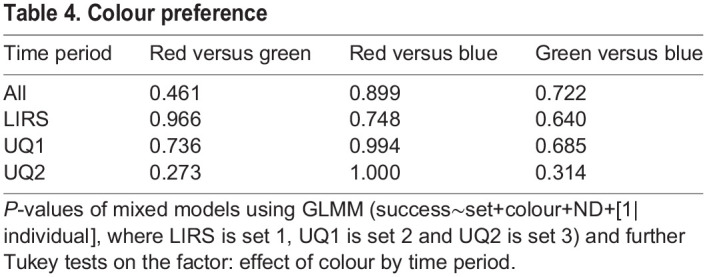
Colour preference

### Experiment 3: naive-choice tests

**Fig. 6.**
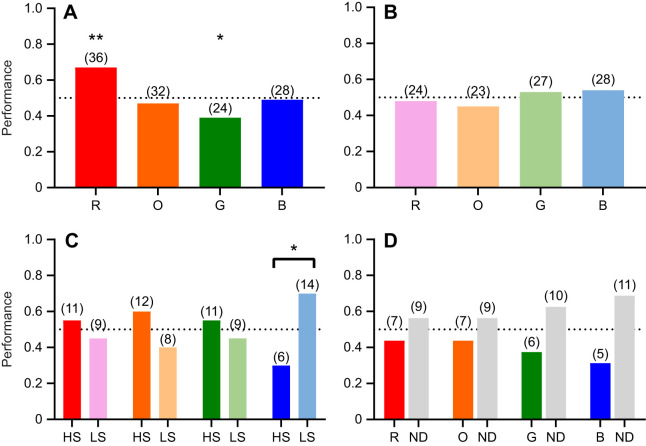
***Haptosquilla trispinosa* naive-choice tests**. (A,B) Results from a 2-choice test between each colour at (A) high saturation and (B) low saturation (i.e. R/O, R/G, R/B, O/G, O/B, G/B). (C) Choice between high-saturation (HS) and low-saturation (LS) stimuli of the same colour. (D) Choice between a high-saturation colour (R, O, G, B) and ND grey (ND 0, 0.15, 0.6, 0.9). The dotted black line indicates the chance level. Numbers in parentheses refer to the number of individuals with the respective preference. See Fig. 5 for significance explanation.

*Haptosquilla trispinosa* were given a 2-choice test to determine whether there was an innate preference for any of the colours or colour types. There was a significant preference for red (binomial test, *P*<0.01) and aversion to green (*P*<0.05) among highly saturated colours (Fig. 6A). No preference was found for any low-saturation colours (Fig. 6B; Table S2).

Other individuals were given a choice between the high- and low-saturation stimuli of each colour. Animals preferred low-saturation to high-saturation blue (*P*<0.05) but had no preference between saturation types for the other colours (Fig. 6C; Table S2). When stomatopods were given a choice between a colour filter and ND filter, they preferred the ND filter to all colours (*P*<0.05), but this was not significant when colours were analysed separately (Fig. 6D). However, they significantly avoided the brightest ND filter (ND 0, *P*<0.05) (Fig. S1B).

## DISCUSSION

The aim of this study was to examine colour processing in *H. trispinosa*, by exploring the two current hypotheses, opponent processing and pattern activation (also called barcode or cochlea-vision; Marshall et al., 2007). Our initial hypotheses were that stomatopods may: (a) fail at all low-saturation colours (mirroring and extending the results of Marshall et al., 1996, and Thoen et al., 2014) or (b) just fail with low-saturation blue, suggesting some behavioural relevance or significance for this colour. This would have provided evidence, albeit circumstantial, that their colour processing was somehow different and potentially barcode like. It should be noted that Marshall et al. (1996) did not exclude opponent processing for colour vision, pointing out that the chromatic signal differential was lowest for the low-saturation blue which their study species (*O. scyllarus*) failed on.

The results of our study did not clarify this debate in stomatopod vision (Marshall and Arikawa, 2014; Marshall et al., 1996, 2007; Thoen et al., 2014). Interestingly, some of our findings raise questions relating to the methodologies in previous and current experiments regarding animal colour vision capabilities. Hence the reason for conducting experiments 2 and 3 here, as discussed below.

We found that *H. trispinosa* could learn to discriminate both high- and low-saturation colours from grey. This was an unexpected result as we originally proposed that all low-saturation colours would be harder to discriminate based on the results of Marshall et al. (1996) and Thoen et al. (2014) (Fig. 5A). *Haptosquilla trispinosa* demonstrated relatively poor discrimination of high-saturation orange and blue colours, compared with other colours; however, we suggest this was due to length of time spent in captivity, rather than being an accurate reflection of their visual capabilities. Extended time in captivity may reduce visual capacity as a result of the light spectrum being more limited than natural daylight, or possibly other influences such as a restricted diet (Fig. 5B). Most notably, stomatopods require carotenoids for the formation of red filters in the retina, and these must be obtained through their diet (Cronin and Caldwell, 2002; Marshall et al., 1991b). Additionally, there was significantly less participation over time in captivity, suggesting a possible loss of motivation.

In light of this, the previous finding that *O. scyllarus* could not discriminate low-saturation blue may be a product of spending time in captivity. In particular, because of the assumption that if humans can see the colour, another animal with apparently superior colour vision must be able to, little effort was made to imitate natural daylight. Animals were kept in an inside room with no window and whatever fluorescent strip-light was present in the room at the time. Furthermore, as animals were sourced from a tropical marine supplier, the amount of time in captivity prior to experiments starting was unknown. An alternative explanation for the apparently contradictory results we obtained is one of species specificity. Blue and indeed other low-saturation colours may be more salient and important for *H. trispinosa* than for *O. scyllarus*.

Since 1996, it has been demonstrated that the spectral sensitivities of stomatopods, and those of other animals, are remarkably plastic, being influenced by both depth and light environment, as well as changing seasonally or on even shorter time scales, such as days or within a single day (crustaceans: Cronin et al., 2002; Jessop et al., 2020; fishes, review: Carleton et al., 2020; Musilova et al., 2021). Our results with *H. trispinosa* further support this visual plasticity, and suggest it is possible that the animals trained in 1996 had a short-term modified colour sense significantly different to the one ordinarily present under natural lighting conditions (Fig. 5). Even in the wild, stomatopods are known to modify and tune spectral sensitivity depending on the spectral envelope of ambient light they live in, according to the depth of habitat they settle into as post-larvae (Cronin and Caldwell, 2002; Cronin et al., 2000; 2001; Cheroske et al., 2006; Osorio et al., 1997).

The results of experiment 1 led to the conception of experiment 2, to determine whether the previous findings of Marshall et al. (1996) could be explained by time in captivity and/or lighting conditions. Interestingly, stomatopods had trouble distinguishing highly saturated red and blue after 3 months in captivity (Fig. 5B), supporting the idea that the negative result of Marshall et al. (1996) needs to be viewed with caution or may indeed be wrong. More recently, we have discovered that mantis shrimps are able to shift their spectral sensitivity under different light environments, both natural and unnatural, and do so on the same time scales seen here (Cheroske et al., 2003, 2006; Cronin et al., 2000). This shift is usually towards shorter wavelengths in the more red-sensitive row 3, an adaptative response to the reduced long wavelengths in deeper or bluer habitats (Cheroske et al., 2006; Cronin et al., 2002; Cronin and Caldwell, 2002). The result here suggests that the stomatopods used in Marshall et al. (1996) had a change in colour discrimination ability and that the failure to discriminate unsaturated blue from grey may have been a result of this short-term adaptation process.

In response to the results from experiment 1 – where *H. trispinosa* displayed a reduced ability to learn saturated orange and blue – we also conducted experiment 3 to test whether *H. trispinosa* has an innate avoidance or preference for colours used in this experiment. *Haptosquilla trispinosa* display bright blue markings on the carapace and maxillipeds in aggressive and mating contexts (Chiou et al., 2005), making it possible that blue may have a specific significance attached to it. This and other species have been found to spontaneously avoid UV markings, also a feature of the frontal displays of stomatopods when they meet or contest ownership of cavities within which to live on the reef (Bok et al., 2018). Our results indicate that *H. trispinosa* did not show any specific avoidance of highly saturated blue but did display a preference for red and an avoidance of green (Fig 6A; Fig. S2A). The preference for longer wavelengths is similar to the results of Daly et al. (2017) where a colour preference for yellow and an avoidance of red was found in naive choice tests in *O. scyllarus*. The nature of the tests conducted was different, but this comparison highlights a potential difference in colour behaviour between species. In addition, we found that *H. trispinosa* had no preference among low-saturation colours (Fig. 6B), but did prefer low- over high-saturation blue (Fig. 6C). This may account for the difference in success rate between high- and low-saturation blue in experiment 1 and may indicate an underlying avoidance of blue in this species (Fig. 5A).

Different specific colour preferences in naive choice tests have been found in other crustaceans. For example, male blue crabs, *Callinectes sapidus*, show a preference for red over orange claws in females, as mature females have red claws while those of prepubertal females are orange (Baldwin and Johnsen, 2012). On the other end of the spectrum, fiddler crab (*Uca mjoebergi*) females prefer males whose yellow claws are also UV reflective (Detto and Backwell, 2009). Among insects, innate long-wavelength preference also occurs in some species of butterflies (Kinoshita and Arikawa, 2014; Swihart and Swihart, 1970; Weiss, 1997) and flower-pollinating flies (An et al., 2018; Lunau et al., 2018).

Interestingly, naive-choice tests showed that *H. trispinosa* had a preference for medium-brightness grey over high-saturation colours (Fig. 6D). Conversely, innate preference tests in crabs show that they prefer colours over grey, which is suggested to be due to the use of colour in sexual selection. Female fiddler crabs prefer yellow over grey, similar to male claw coloration (Detto, 2007), while male blue crabs prefer red claws, which mature females exhibit, over white and black claws (Baldwin and Johnsen, 2009).

To find out whether the ability to distinguish both low- and high-saturation colours is more widespread among stomatopods, we repeated experiments 1 and 2 with *Gonodactylus smithii.* In common with *H. trispinosa*, there was an overall trend towards learning low-saturation colours (*P*<0.05), and a degradation of performance over time kept in captivity (*P*<0.001; Fig. S2, Table S3).

Although the set of experiments described here have further explored the colour discrimination of stomatopods, even the basics of the colour processing mechanism remain unclear. It is possible that stomatopods use a combination of multiple mechanisms to process colour information in different behavioural contexts, including opponency and photoreceptor activation comparisons, or barcode analysis. In addition, although the retinal design and underlying structures are largely similar in all mantis shrimp species with six-row midbands, different species may process colour differently (Marshall et al., 2007; Thoen et al., 2017).

Given that mantis shrimps display species-specific colour markings during encounters between and within species on the reef (Caldwell and Dingle, 1975), it is worth considering whether this aspect of behaviour plays a stronger part in stomatopod colour vision and that the colour of food, or food containers, is irrelevant in normal life. During aggression sequences, often both mantis shrimp spread their front raptors to show the species-specific colour of their meral spot to evaluate their opponent – an act that may lead to either fighting or submission (Caldwell and Dingle, 1975; Dingle and Caldwell, 1969; Green and Patek, 2015; 2018). Both the intensity and chromaticity of the meral spot are a signal of aggression in some species: a darker meral spot indicates a stronger strike force, and a lighter meral spot often leads to the receiver increasing antagonism (Caldwell and Dingle, 1975; Franklin et al., 2017; 2019; Hazlett, 1979).

While the results of the experiments here have not led to firm conclusions, what is clear is that a number of previous experimental protocols in colour vision experimentation may need adjusting. The fact that vision changes over evolutionarily short time spans (reviewed in Land and Nilsson, 2012; Nilsson, 2013; Kelber and Osorio, 2010; Marshall et al., 2015) and that, apparently, colour vision is remarkably plastic in both vertebrates (Carleton et al., 2020; Kelber et al., 2003; Musilova et al., 2021) and invertebrates (Cronin et al., 2002; Jessop et al., 2020; Strausfeld and Andrew, 2011; van der Kooi et al., 2020) on very short time scales presents a fascinating area for further study. Does a change in visual pigment expression level or photoreceptor complement lead to a change in colour detection or discrimination? What degree of colour constancy underlies these changes? Is a food reward-based behavioural experiment sufficiently basal that other colour-based behaviours, such as mate choice or aggressive interaction, simply follow suit, or are there different levels of discrimination for different behavioural tasks? As usual with the stomatopods, we have found more questions than answers.

## Supplementary Material

Supplementary information

## References

[JEB243699C1] An, L., Neimann, A., Eberling, E., Algora, H., Brings, S. and Lunau, K. (2018). The yellow specialist: Dronefly Eristalis tenax prefers different yellow colours for landing and proboscis extension. *J. Exp. Biol.*, 221, jeb184788. 10.1242/jeb.184788 30190319

[JEB243699C2] Arikawa, K. (2003). Spectral organization of the eye of a butterfly, Papilio. *Journal of Comparative Physiology A: Neuroethology, Sensory, Neural, and Behavioral Physiology* 189, 791-800. 10.1007/s00359-003-0454-7 14520495

[JEB243699C3] Baldwin, J. and Johnsen, S. (2009). The importance of color in mate choice of the blue crab Callinectes sapidus. *J. Exp. Biol.* 212, 3762-3768. 10.1242/jeb.028027 19880739

[JEB243699C4] Baldwin, J. and Johnsen, S. (2012). The male blue crab, Callinectes sapidus, uses both chromatic and achromatic cues during mate choice. *J. Exp. Biol.* 215, 1184-1191. 10.1242/jeb.067512 22399664

[JEB243699C5] Barlow, H. B. (1982). What causes trichromacy? A theoretical analysis using comb-filtered spectra. *Vision Res.* 22, 635-643. 10.1016/0042-6989(82)90099-2 6981244

[JEB243699C6] Bates, D., Mächler, M., Bolker, B. M. and Walker, S. C. (2015). Fitting linear mixed-effects models using lme4. *J. Stat. Softw.*, 67, 1-48. 10.18637/jss.v067.i01

[JEB243699C7] Bok, M. J., Porter, M. L., Place, A. R. and Cronin, T. W. (2014). Biological sunscreens tune polychromatic ultraviolet vision in mantis shrimp. *Curr. Biol.* 24, 1636-1642. 10.1016/j.cub.2014.05.071 24998530

[JEB243699C8] Bok, M. J., Roberts, N. W. and Cronin, T. W. (2018). Behavioural evidence for polychromatic ultraviolet sensitivity in mantis shrimp. *Proc. R. Soc. B*, 285, 10.1098/rspb.2018.1384 PMC6111172 30068672

[JEB243699C9] Caldwell, R. L. and Dingle, H. (1975). Ecology and evolution of agonistic behavior in stomatopods. *Naturwissenschaften* 62, 214-222. 10.1007/BF00603166

[JEB243699C10] Carleton, K. L., Escobar-Camacho, D., Stieb, S. M., Cortesi, F. and Marshall, N. J. (2020). Seeing the rainbow: mechanisms underlying spectral sensitivity in teleost fishes. *J. Exp. Biol.* 223, jeb193334. 10.1242/jeb.193334 32327561 PMC7188444

[JEB243699C11] Chen, P.-J., Awata, H., Matsushita, A., Yang, E.-C. and Arikawa, K. (2016). Extreme spectral richness in the eye of the common bluebottle butterfly, Graphium sarpedon. *Frontiers in Ecology and Evolution* 4, 1-12. 10.3389/fevo.2016.00018

[JEB243699C12] Cheroske, A. G., Cronin, T. W. and Caldwell, R. L. (2003). Adaptive color vision in Pullosquilla litoralis (Stomatopoda, Lysiosquilloidea) associated with spectral and intensity changes in light environment. *J. Exp. Biol.* 206, 373-379. 10.1242/jeb.00084 12477907

[JEB243699C13] Cheroske, A. G., Barber, P. H. and Cronin, T. W. (2006). Evolutionary variation in the expression of phenotypically plastic color vision in Caribbean mantis shrimps, genus Neogonodactylus. *Marine Biology* 150, 213-220. 10.1007/s00227-006-0313-5

[JEB243699C14] Chiou, T.-H., Cronin, T. W., Caldwell, R. L. and Marshall, J. (2005). Biological polarized light reflectors in stomatopod crustaceans. *Polarization Science and Remote Sensing II* 5888, 58881B. 10.1117/12.613117

[JEB243699C15] Cronin, T. W. and Caldwell, R. L. (2002). Tuning of photoreceptor function in three mantis shrimp species that inhabit a range of depths II. Filter pigments. *J Comp Physiol A* 188, 187-197. 10.1007/s00359-002-0292-z 11976886

[JEB243699C16] Cronin, T. W. and Marshall, N. J. (1989). Multiple spectral classes of photoreceptors in the retinas of gonodactyloid stomatopod crustaceans. *Journal of Comparative Physiology A* 166, 261-275. 10.1007/BF00193471

[JEB243699C17] Cronin, T. W., Marshall, N. J. and Caldwell, R. L. (2000). Spectral tuning and the visual ecology of mantis shrimps. *Philosophical Transactions of the Royal Society B: Biological Sciences* 355, 1263-1267. 10.1098/rstb.2000.0680 PMC1692847 11079411

[JEB243699C18] Cronin, T. W., Caldwell, R. L. and Marshall, N. J. (2001). Sensory adaptation. Tunable colour vision in a mantis shrimp. *Nature* 411, 547-548. 10.1038/35079184 11385560

[JEB243699C19] Cronin, T. W., Caldwell, R. L. and Erdmann, M. V. (2002). Tuning of photoreceptor function in three mantis shrimp species that inhabit a range of depths I. Visual pigments. *J Comp Physiol A* 188, 179-186. 10.1007/s00359-002-0291-0 11976885

[JEB243699C20] Daly, I. M., Tetley, A. E., Jared, S. L., How, M. J. and Roberts, N. W. (2017). Colour preference in Odontodactylus scyllarus (Linnaeus, 1758) (Stomatopoda). *J. Crustac. Biol.* 37, 374-379. 10.1093/jcbiol/rux038

[JEB243699C21] Detto, T. (2007). The fiddler crab Uca mjoebergi uses colour vision in mate choice. *Proc. R. Soc. B* 274, 2785-2790. 10.1098/rspb.2007.1059 PMC3227134 17848366

[JEB243699C22] Detto, T. and Backwell, P. R. Y. (2009). The fiddler crab Uca mjoebergi uses ultraviolet cues in mate choice but not aggressive interactions. *Anim. Behav.* 78, 407-411. 10.1016/j.anbehav.2009.05.014

[JEB243699C23] Dingle, H. and Caldwell, R. (1969). The aggressive and territorial behaviour of the mantis shrimp Gonodactylus bredini Manning (Crustacea: Stomatopoda). *Behaviour* 33, 115-136. 10.1163/156853969X00341 5815890

[JEB243699C24] Franklin, A. M., Applegate, M. B., Lewis, S. M. and Omenetto, F. G. (2017). Stomatopods detect and assess achromatic cues in contests. *Behav. Ecol.* 28, 1329-1336. 10.1093/beheco/arx096

[JEB243699C25] Franklin, A. M., Donatelli, C. M., Culligan, C. R. and Tytell, E. D. (2019). Meral-spot reflectance signals weapon performance in the mantis shrimp neogonodactylus oerstedii (Stomatopoda). *Biological Bulletin* 236, 43-54. 10.1086/700836 30707606

[JEB243699C26] Glantz, R. M. and Miller, C. S. (2002). Signal processing in the crayfish optic lobe: contrast, motion and polarization vision. In *The Crustacean Nervous System* (ed. K. Wiese), pp. 486-498. Springer.

[JEB243699C27] Green, P. A. and Patek, S. N. (2015). Contests with deadly weapons: Telson sparring in mantis shrimp (Stomatopoda). *Biol. Lett.*, 11, 10.1098/rsbl.2015.0558 PMC4614432 26399976

[JEB243699C28] Green, P. A. and Patek, S. N. (2018). Mutual assessment during ritualized fighting in mantis shrimp (Stomatopoda). *Proceedings. Biological Sciences* 285, 20172542. 10.1098/rspb.2017.2542 29343603 PMC5805947

[JEB243699C29] Hazlett, B. (1979). The meral spot of Gonodactylus oerstedii Hansen as a visual stimulus (Stomatopoda, Gonodactylidae). *Crustaceana* 36, 196-198. 10.1163/156854079X00429

[JEB243699C63] Hothorn, T., Bretz, F. and Westfall, P. (2008). Simultaneous inference in general parametric models. *Biom. J.* 50, 346-363. 10.1002/bimj.200810425 18481363

[JEB243699C30] Jessop, A. L., Ogawa, Y., Bagheri, Z. M., Partridge, J. C. and Hemmi, J. M. (2020). Photoreceptors and diurnal variation in spectral sensitivity in the fiddler crab Gelasimus dampieri. *J. Exp. Biol.*, 223, jeb230979. 10.1242/jeb.230979 33097568

[JEB243699C31] Kelber, A. and Osorio, D. (2010). From spectral information to animal colour vision: Experiments and concepts. *Proc. R. Soc. B* 277, 1617-1625. 10.1098/rspb.2009.2118 PMC2871852 20164101

[JEB243699C32] Kelber, A., Vorobyev, M. and Osorio, D. (2003). Animal colour vision – behavioural tests and physiological concepts. *Biol. Rev. Camb. Philos. Soc.* 78, 81-118. 10.1017/S1464793102005985 12620062

[JEB243699C33] Kinoshita, M. and Arikawa, K. (2014). Color and polarization vision in foraging Papilio. *Journal of Comparative Physiology A: Neuroethology, Sensory, Neural, and Behavioral Physiology* 200, 513-526. 10.1007/s00359-014-0903-5 24722674

[JEB243699C34] Land, M. F. (1999). Motion and vision: why animals move their eyes. *Journal of Comparative Physiology A* 185, 341-352. 10.1007/s003590050393 10555268

[JEB243699C35] Land, M. F. and Nilsson, D. E. (2012). *Animal Eyes*. Oxford, UK: Oxford University Press.

[JEB243699C36] Land, M. F., Marshall, J. N., Brownless, D. and Cronin, T. W. (1990). The eye-movements of the mantis shrimp Odontodactylus scyllarus (Crustacea: Stomatopoda). *Journal of Comparative Physiology A* 167, 155-166. 10.1007/BF00188107

[JEB243699C37] Lunau, K., An, L., Donda, M., Hohmann, M., Sermon, L. and Stegmanns, V. (2018). Limitations of learning in the proboscis reflex of the flower visiting syrphid fly Eristalis tenax. *PLoS ONE* 13, 1-20. 10.1371/journal.pone.0194167 PMC5860702 29558491

[JEB243699C38] Marshall, N. J. (1988). A unique colour and polarization vision system in mantis shrimps. *Nature* 333, 557-560. 10.1038/333557a0 3374602

[JEB243699C39] Marshall, N. J. and Arikawa, K. (2014). Unconventional colour vision. *Curr. Biol.* 24, R1150-R1154. 10.1016/j.cub.2014.10.025 25514002

[JEB243699C40] Marshall, N. J. and Land, M. F. (1993). Some optical-features of the eyes of Stomatopods.2. Ommatidial design, sensitivity and habitat. *Journal of Comparative Physiology A* 173, 583-594. 10.1007/BF00197766

[JEB243699C41] Marshall, N. J. and Vorobyev, M. (2003). The design of color signals and color vision in fishes. *Sensory Processing in Aquatic Environments* 194-222. 10.1007/978-0-387-22628-6_10

[JEB243699C42] Marshall, N. J., Land, M. F., King, C. and Cronin, T. W. (1991a). The compound eyes of mantis shrimps (Crustacea, Hoplocarida, Stomatopoda) I. Compound eye structure: the detection of polarized light. *Philosophical Transactions of the Royal Society B: Biological Sciences* 334, 33-56. 10.1098/rstb.1991.0096

[JEB243699C43] Marshall, N. J., Land, M. F., King, C. A. and Cronin, T. W. (1991b). The compound eyes of mantis shrimps (Crustacea, Hoplocarida, Stomatopoda). II. Colour pigments in the eyes of Stomatopod Crustaceans: polychromatic vision by serial and lateral filtering. *Philosophical Transactions of the Royal Society B: Biological Sciences* 334, 57-84. 10.1098/rstb.1991.0097

[JEB243699C44] Marshall, N. J., Jones, J. P. and Cronin, T. W. (1996). Behavioural evidence for colour vision in stomatopod crustaceans. *Journal of Comparative Physiology A* 179, 473-481. 10.1007/BF00192314

[JEB243699C45] Marshall, N. J., Cronin, T. W. and Kleinlogel, S. (2007). Stomatopod eye structure and function: A review. *Arthropod. Struct. Dev.* 36, 420-448. 10.1016/j.asd.2007.01.006 18089120

[JEB243699C47] Marshall, N. J., Carleton, K. L. and Cronin, T. W. (2015). Colour vision in marine organisms. *Curr. Opin. Neurobiol.* 34, 86-94. 10.1016/j.conb.2015.02.002 25725325

[JEB243699C48] Musilova, Z., Salzburger, W. and Cortesi, F. (2021). The visual opsin gene repertoires of teleost fishes: evolution, ecology, and function. *Annu. Rev. Cell Dev. Biol.* 37, 1-28. 10.1146/annurev-cellbio-120219-024915 34351785

[JEB243699C49] Nilsson, D. E. (2013). Eye evolution and its functional basis. *Vis. Neurosci.* 30, 5-20. 10.1017/S0952523813000035 23578808 PMC3632888

[JEB243699C50] Osorio, D., Marshall, N. J. and Cronin, T. W. (1997). Stomatopod photoreceptor spectral tuning as an adaptation for colour constancy in water. *Vision Res.* 37, 3299-3309. 10.1016/S0042-6989(97)00136-3 9425545

[JEB243699C51] Strausfeld, N. J. and Andrew, D. R. (2011). A new view of insect-crustacean relationships I. Inferences from neural cladistics and comparative neuroanatomy. *Arthropod. Struct. Dev.* 40, 276-288. 10.1016/j.asd.2011.02.002 21333750

[JEB243699C52] Strausfeld, N. J. and Nassel, D. R. (1981). Neuroarchitectures Serving Compound Eyes of Crustacea and Insects. In *Handbook of Sensory Physiology*, vol. VII/6B (ed. H. Autrum), pp. 34-41. Springer.

[JEB243699C53] Swihart, C. A. and Swihart, S. L. (1970). Colour selection and learned feeding preferences in the butterfly, Linn. *Animal Behaviour* 18, 60-64. 10.1016/0003-3472(70)90071-0

[JEB243699C54] Templin, R. M. (2017). Circular polarization vision in stomatopod crustaceans. *PhD thesis*, University of Queensland, Brisbane, Australia.

[JEB243699C55] Thoen, H. H., How, M. J., Chiou, T.-H. H. and Marshall, J. (2014). A different form of color vision in mantis shrimp. *Science* 343, 411-413. 10.1126/science.1245824 24458639

[JEB243699C56] Thoen, H. H., Strausfeld, N. J. and Marshall, J. (2017). Neural organization of afferent pathways from the stomatopod compound eye. *J. Comp. Neurol.* 525, 36-80. 10.1002/cne.24256 28577301

[JEB243699C57] Thoen, H. H., Sayre, M. E., Marshall, N. J. and Strausfeld, N. J. (2018). Representation of the stomatopod's retinal midband in the optic lobes: Putative neural substrates for integrating chromatic, achromatic and polarization information. *J. Comp. Neurol.* 526, 1148-1165. 10.1002/cne.24398 29377111

[JEB243699C58] van der Kooi, C. J., Stavenga, D. G., Arikawa, K., Belušič, G. and Kelber, A. (2021). Evolution of insect color vision: from spectral sensitivity to visual ecology. *Annu. Rev. Entomol.* 66, 1-28. 10.1146/annurev-ento-061720-071644 32966103

[JEB243699C59] Von Frisch, K. (1974). Decoding the language of the bee. *Science* 185, 663-668. 10.1126/science.185.4152.663 17736362

[JEB243699C60] Weiss, M. R. (1997). Innate colour preferences and flexible colour learning in the pipevine swallowtail. *Anim. Behav.* 53, 1043-1052. 10.1006/anbe.1996.0357

[JEB243699C61] Wolpert, H. D. (2011). An eye toward multispectral imaging. *Optics and Photonics* 22, 16-19.

[JEB243699C62] Zaidi, Q., Marshall, J., Thoen, H. and Conway, B. R. (2014). Evolution of neural computations: Mantis shrimp and human color decoding. *I-Perception* 5, 492-496. 10.1068/i0662sas 26034560 PMC4441025

