# Supplementary material for: Colour vision in stomatopod crustaceans: more questions than answers
Source: J Exp Biol. 2022 Mar 28;225(6):jeb243699. doi: 10.1242/jeb.243699 (PMC9001920; doi:10.1242/jeb.243699)
Supplement: Supplementary information [file jexbio-225-243699-s1.pdf]

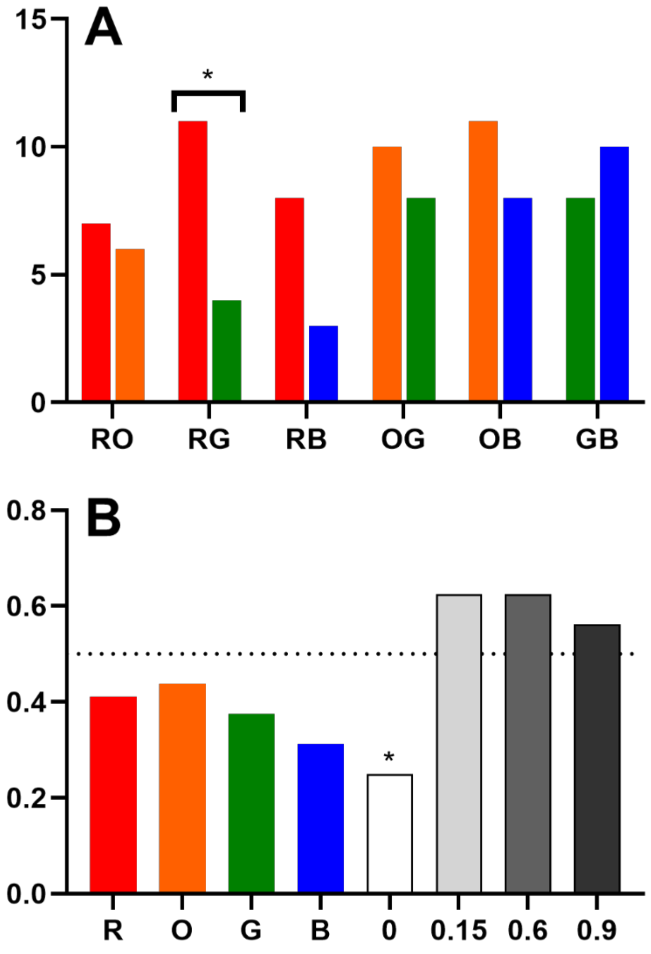

Fig. S1. Details of naïve choice test. A) High saturation choice test with comparison in each choice set. Only when animals were given the option of red or green was there a significant preference. **B)** More detailed results from high saturation colour versus grey preference.

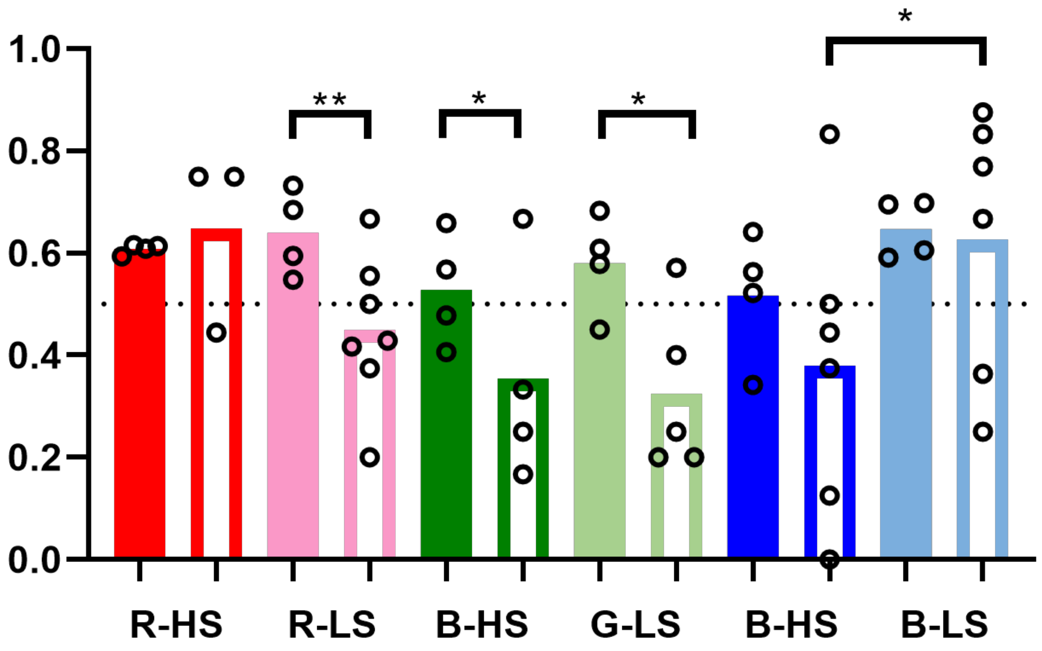

Fig. S2. ***G. smithii*** **performance in captivity.** Average performance of individual in high and low saturation stimulus learning. Comparison between individuals that have been in captivity for 1-2 months (solid bars) versus individuals in captivity for 6-12 months (open bars). Circles represent individual averages. Dotted black line refers to the level of chance.

Table S1. Effect of neutral density distractors. Probability of success in different groups (see methods) with for (**A**) each ND pair in Experiment 1, and (**B**) each ND type in Experiment 2. Italics give significance of difference (p values) using model: Success ~ ND + [1|individual].

**A**		**R**		**O**		**G**		**B**	
	**All**	**HS**	**LS**	**HS**	**LS**	**HS**	**LS**	**HS**	**LS**
**0/0**	0.65<br>0.0001***	0.57<br>0.51	0.74<br>0.0037**	0.36<br>0.37	0.73<br>0.19	0.73<br>0.026*	0.62<br>0.15	0.48<br>0.90	0.75<br>0.0026**
**0/0.15**	0.51<br>0.00345**	0.47<br>0.40	0.49<br>0.025*	0.63<br>0.27	0.60<br>0.55	0.45<br>0.017*	0.50<br>0.32	0.30<br>0.15	0.79<br>0.68
**0/0.6**	0.57<br>0.0700	0.52<br>0.59	0.67<br>0.50	0.38<br>0.95	0.50<br>0.26	0.50<br>0.037*	0.63<br>0.88	0.42<br>0.62	0.75<br>1.0
**0/0.9**	0.58<br>0.142	0.45<br>0.30	0.52<br>0.047*	0.62<br>0.22	0.76<br>0.75	0.56<br>0.20	0.52<br>0.39	0.61<br>0.38	0.68<br>0.50
**0.15/0.15**	0.38<br>4.6e-10***	0.30<br>0.011*	0.43<br>0.0038**	0.29<br>0.62	0.42<br>0.092	0.35<br>0.0007***	0.46<br>0.15	0.30<br>0.10	0.50<br>0.019*
**0.15/0.6**	0.43<br>1.8e-7***	0.42<br>0.14	0.44<br>0.0066**	0.40<br>0.85	0.42<br>0.093	0.45<br>0.0080**	0.50<br>0.27	0.39<br>0.50	0.38<br>0.00086***
**0.15/0.9**	0.42<br>5.4e-6***	0.32<br>0.049*	0.45<br>0.027*	0.36<br>0.98	0.48<br>0.20	0.42<br>0.022*	0.35<br>0.036*	0.29<br>0.36	0.67<br>0.49
**0.6/0.6**	0.43<br>4.9e-7***	0.28<br>0.0074**	0.50<br>0.024*	0.36<br>0.97	0.44<br>0.13	0.45<br>0.014*	0.45<br>0.14	0.35<br>0.18	0.61<br>0.20
**0.6/0.9**	0.54<br>0.0373*	0.50<br>0.54	0.58<br>0.16	0.29<br>0.73	0.50<br>0.28	0.62<br>0.42	0.62<br>1.0	0.44<br>0.24	0.57<br>0.16
**0.9/0.9**	0.62<br>0.618	0.67<br>0.42	0.67<br>0.50	0.48<br>0.54	0.38<br>0.078	0.63<br>0.43	0.69<br>0.53	0.53<br>0.85	0.77<br>0.81

**B**	**All**	**R**	**G**	**B**	**Set 1 (LIRS)**	**Set 2**	**Set 3**
**0**	0.61<br>2.65e-5***	0.59<br>0.0504	0.66<br>0.00141**	0.61<br>0.0122*	0.61<br>0.0204*	0.73<br>5.26e-6***	0.43<br>0.642
**0.15**	0.58<br>0.0538	0.54<br>0.224	0.67<br>0.548	0.56<br>0.121	0.54<br>0.257	0.63<br>0.102	0.52<br>0.194
**0.6**	0.58<br>0.0097**	0.55<br>0.222	0.60<br>0.256	0.58<br>0.0242*	0.49<br>0.0564	0.65<br>0.0401*	0.51<br>0.348
**0.9**	0.53<br>0.914	0.47<br>0.686	0.45<br>0.561	0.66<br>0.282	0.54<br>0.625	0.57<br>0.596	0.50<br>0.995

Table S2. Preference in naïve two-choice tests. Numbers refer to the amount of individuals that chose each option. **A)** Tests comparing two high saturation colours. **B)** Tests comparing two low saturation colours. **C)** Tests comparing high and low saturation types of each colour. **D)** Tests comparing a high saturation colour and a neutral grey. Significance calculated using a binomial test.

**A**		**HS**		**Total**	***p-value***
	R	36		54	0.0054 **
	O	32		68	0.086
	G	24		61	0.026 *
	B	28		57	0.104
**B**		**LS**			
	R	24		50	0.107
	O	23		51	0.087
	G	27		51	0.102
	B	28		52	0.095
**C**		**HS**	**LS**		
	R	11	9	20	0.160
	O	12	8	20	0.120
	G	11	9	20	0.160
	B	6	14	20	0.037 *
**D**		**Colour**	**ND**		
	R	7	9	16	0.174
	O	7	9	16	0.174
	G	6	10	16	0.122
	B	5	11	16	0.067

Journal of Experimental Biology • Supplementary information

Table S3. Number of individuals and average participation per colour type per set in *G. smithii*. Average success, standard error, and learning ability calculated as a binomial (see methods). *P-values* for high and low saturation performance in set 1 (short term) as well as from comparison of colour types between set 1 and set 2 (far right column).

†Model: Success ~ Type + ND + [1|individual]

‡Model: Success ~ Set + ND + [1|individual]

	Colour	Type	Individuals	Participation	Average % Success ±SE	Learning Ability	Compare Type†	Compare to Short-Term‡
Short-Term	R	HS	4	34.5	60.8 ±0.5	0.0026 **	0.717	
		LS		35	64.0 ±4.2	0.0005 ***		
	G	HS		36	52.8 ±5.5	0.0370 *	0.317	
		LS		26	58.0 ±4.9	0.0093 **		
	B	HS		39	51.7 ±6.3	0.0591	0.0104 *	
		LS		42	64.7 ±2.9	< 0.0001 ***		
Long-Term	R	HS	3	5.7	64.8 ±10.2	0.1484		0.871
		LS	7	8.9	44.9 ±5.7	0.0758		0.0057 **
	G	HS	4	5.5	35.4 ±11.0	0.0762 *		0.0293 *
		LS	5	5.2	32.4 ±7.2	0.0466 *		0.0108 *
	B	HS	6	6.5	38.0 ±12.0	0.0457 *		0.273
		LS	6	8	62.6 ±10.6	0.0151 *		0.824

Journal of Experimental Biology • Supplementary information
